# (Tn5-)FISH-based imaging in the era of 3D/spatial genomics

**DOI:** 10.52601/bpr.2023.220025

**Published:** 2023-02-28

**Authors:** Liheng Yang, Yan Yan, JunLin Li, Cheng Zhou, Jinlan Jin, Tongmei Zhang, Haokaifeng Wu, Xingang Li, Wei Wang, Li Yuan, Xu Zhang, Juntao Gao

**Affiliations:** 1 Seaver College, Pepperdine University, CA 90263, USA; 2 Center for Synthetic & Systems Biology, Tsinghua University, Beijing 100084, China; 3 Bioinformatics Division, BNRist, Department of Automation, Beijing 100084, China; 4 MOE Key Laboratory of Bioinformatics, Beijing 100084, China; 5 Savaid Medical School, University of Chinese Academy of Sciences, Beijing 100084, China; 6 Department of Radiation Oncology, Nanfang Hospital, Southern Medical University, Guangzhou 510515, China; 7 Department of Critical Care Medicine, Shenzhen Hospital (Futian) of Guangzhou University of Chinese Medicine, Shenzhen, Guangdong 518034, China; 8 Medical Oncology, Beijing Chest Hospital, Capital Medical University & Beijing Tuberculosis and Thoracic Tumor Research Institute, Beijing 101149, China; 9 Centre for Regenerative Medicine and Health, HongKong Institute of Science & Innovation, Chinese Academy of Sciences, HongKong SAR, China; 10 Key Laboratory of Biological Targeting Diagnosis, Therapy and Rehabilitation of Guangdong Higher Education Institutes, The Fifth Affiliated Hospital of Guangzhou Medical University, Guangzhou 510000, China; 11 Beijing Institute of Collaborative Innovation, Beijing 100094, China; 12 Institute for TCM-X, Beijing 100084, China; 13 Centre for Precision Health, Edith Cowan University, Perth, WA 6027, Australia

**Keywords:** Tn5-FISH, Molecular imaging, Chromatin interaction, Regulatory elements

## Abstract

3D genomics mainly focuses on the 3D position of single genes at the cell level, while spatial genomics focuses more on the tissue level. In this exciting new era of 3D/spatial genomics, half-century old FISH and its derivative methods, including Tn5-FISH, play important roles. In this review, we introduce the Tn5-FISH we developed recently, and present six different applications published by our collaborators and us, based on (Tn5-)FISH, which can be either general BAC clone-based FISH or Tn5-FISH. In these interesting cases, (Tn5-)FISH demonstrated its vigorous ability of targeting sub-chromosomal structures across different diseases and cell lines (leukemia, mESCs (mouse embryonic stem cells), and differentiation cell lines). Serving as an effective tool to image genomic structures at the kilobase level, Tn5-FISH holds great potential to detect chromosomal structures in a high-throughput manner, thus bringing the dawn for new discoveries in the great era of 3D/spatial genomics.

## INTRODUCTION

3D genomics mainly focuses on the 3D position of single genes at the single cell level, while spatial genomics focuses more on the tissue level, and pays more attention to the spatial position of different cells in one tissue.

The spatial architecture of the genome could greatly affect the genomic function. Various genomic architectures such as chromatin loops, domains, compartments, and lamina, along with nucleolus-associated regions, have been widely studied. However, the relationship between spatial organizations and gene regulation in various cell types in mammalian cells is not yet fully understood.

Hi-C (Dekker and Mirny 2016; Lieberman-Aiden *et al*. 2009) and its derivative methods demonstrated two types of the intermediate structure known as topological associating domains (TADs) and A/B compartments (Dixon *et al*. 2012). It remains largely unknown how these genomic architectures are jointly organized within the single cells and how these structures correlate with each other across all cell types. Even with the recently developed single-cell Hi-C method (Rowley and Corces 2018), these methods are not capable of profiling the RNA expression and single-cell mapping in an *in situ* manner.

Fluorescence *in situ* hybridization (FISH), enables the detection and localization of a specific non-repetitive DNA sequence visible on a chromosome. The FISH technique avoided hypotonic treatment and methanol-acetic acid dehydrating fixation leading to the enlargement of nuclei and chromatin which skews the measurement between genes (Murmann *et al.* 2005). Therefore, the gene positions could be objectively represented and measured using 3D-FISH. Therefore, FISH keeps being the gold standard technique to detect gene copy number changes and is critical in the diagnosis of cancers and other diseases in clinics.

Nick translation-based traditional BAC-clone FISH (Kim *et al.* 1996), which is based on the typical BAC clone expanding 100–300 kb (Shizuya and Kouros-Mehr 2001), requires a careful balance of DNase and polymerase. The qualities of probes depend on the target region, variations in fragment size, melting temp per fragment, and the amount of background noise produced from repetitive regions.

Furthermore, ~50 kb-long genes locate in the TAD (topologically associated domain) which is usually less than 1 Mb, while the chromatin interactions identified by most 3C (Dekker *et al.* 2002) based techniques fall into the range between 1 kb and 100 kb. Traditional BAC FISH is ineffective to label, or is incapable of imaging the kb-long regulatory elements in these genes.

Therefore, various FISH methods targeted to address this issue were developed during the last decades. For example, Oligopaints, which utilizes PCR primers conjugated with one fluorophore per oligo probe, enables strand-specific or single-strand amplification from kilobases (Chi *et al*. 2005; Danielian *et al.* 1997) to megabases hybridization powered by bountiful oligo library (Beliveau *et al.* 2012, 2018; Schmidt *et al.* 2015).

Other methods with kb resolution, including Tn5-FISH (Tn5 transposase-based fluorescence *in situ *hybridization) (Niu *et al*. 2020), MB-FISH, HD-FISH, *etc*, aim to label genomic loci with kilobase resolution, in order to provide useful tools for the era of spatial genomics. One of these methods, Tn5-FISH, will be introduced in details here.

## HIGHLIGHT OF TN5-FISH

**Figure 1:**
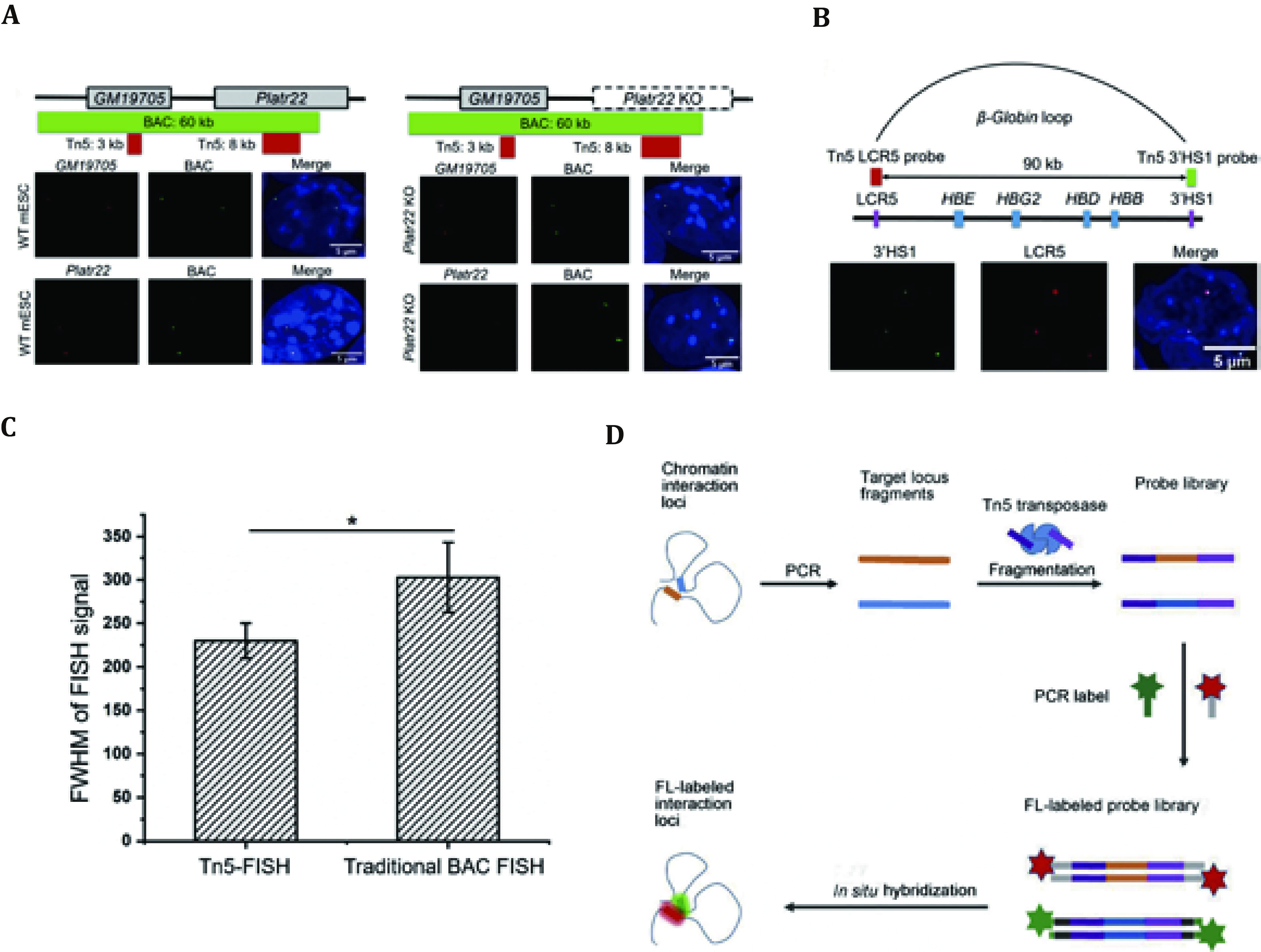
**A** The upper panel demonstrated the BAC FISH probe (green rectangle box) compared with the Tn5-FISH probe (red rectangle box) marked using triple color. The lower panel demonstrated Tn5-FISH (red) and BAC probe (green) in *GM19705* and *Platr22*. **B** The upper panel demonstrated Tn5-FISH probes across the beta-Globin loop (90 kb apart) along with its major components (represented in blue and purple rectangle boxes). The lower panel demonstrated Tn5-FISH 3’HS1 (red) probe and the LCR5 probe (green). **C** The full width at half maximum (FWHM) signal comparison between Tn5-FISH and traditional BAC-FISH. The result suggests that the Tn5-FISH probe is better to resolve smaller genomic components. **D** The schematic demonstration of Tn5-FISH from identification of the chromatin interaction loci to *in situ* hybridization (Distributed under the terms of the Creative Commons CC BY license, Niu *et al.* 2020)

Tn5-FISH, a cost-effective, PCR-based imaging method, used hyperactive transposase to label chromatins several kilobases long, with much higher resolution (one or two orders of magnitude) than most traditional FISH methods. In addition, the hyperactive Tn5 transposase was used for probe library construction, and PCR, which is more cost-effective than probe synthesis, was used for labeling (Fig. 1).

Tn5-FISH also demonstrated its rigorous spatial imaging ability when dealing with short-range chromatin regions which located within the contact domain and TAD. For example, Tn5-FISH was used to image the short-range interactions in the well-known keratin-encoding gene (KRT) locus to demonstrate its ability for validation of short chromatin interactions (Niu *et al*. 2020).

To further validate the effectiveness of Tn5-FISH on labeling sub-chromosomal structures, Tn5-FISH was used to target two adjacent loci: GM19705 and Platr22 that are only 6.5 kb away from each other, in the mm9 reference genome (Niu *et al.* 2020). The GM19705 loci in wild-type mESCs and Platr22-KO mESCs were hybridized to examine Tn5-FISH’s ability to target loci that are merely 6.5 kb with each other. GM19705 locus can be visualized in wild-type mESCs and Platr22-KO mESCs, while Platr22 locus is only visualized in wild-type but not in Platr22-KO mESCs. The result was cross-validated with traditional BAC-based FISH (Fig. 1A), indicating that Tn5-FISH is indeed powerful to label short regions less than 10kb. Since Tn5 probes are smaller, the spatial resolution of Tn5-FISH can be improved to 230 nm, which can be further improved, if necessary, while BAC FISH can only have the spatial resolution of around 300 nm (Niu *et al.* 2020).

Beta globin cluster is used for the third validation. In K562 cells, the interaction between 3’HS1 and LCR5 genomic loci in Beta globin cluster, which is only 90 kb apart from each other, were identified by Hi-C. Tn5-FISH successfully co-localized both 3’HS1 and LCR5. Type II KRT locus in chr12 of K562 measured by Hi-C contact map (Rao *et al.* 2014) is also used to further validate that Tn5-FISH is very versatile for labeling unique genomic loci with high specificity (Fig. 1A).

All of these cases indicated that specific genomic loci and chromatin interaction inside TADS can be visualized by Tn5-FISH, in both normal and cancer cell lines, with traditional BAC-FISH as a positive control.

On the other hand, hyperactive Tn5 offered plasticity in DNA probe template construction in small quantities (1–50 ng, which can be adjusted on commercial availability through Tn5 library construction kits), about 20 times less than BAC clone.

Besides, compared to other PCR-based FISH, Tn5-FISH probe concentration is less, 15–50 ng/μL for suspension cells and 30–50 ng/μL for adherent cells, suggesting that high concentration is not required (although high concentration will be required when labeling the whole or several chromosomes). Therefore, Tn5-FISH lowered the mispriming frequency, dropping the false positive rate.

In addition, sequencing bias is significantly reduced in Tn5-FISH since only a very short genomic fragment is needed for amplification. The probes generated by Tn5-FISH has a higher labeling density than those by nick translation.

Last but not the least, Tn5-FISH can be used to label genomic loci in MANY, if not all, species, while BAC FISH is limited by only two species, mouse and human.

### Six applications for TN5-FISH or FISH

Here we demonstrate six applications published by us and our collaborators, to show how (Tn5-)FISH (which can be either Tn5-FISH or FISH) method contributes to the study of 3D genomics in leukemia, embryonic stem cells (ESCs) and differentiated cell lines.

#### (Tn5-)FISH application in mESCs

CTCF, an architecture protein used to mediate chromatin loops, is found to be organizing long-range chromatin interaction in Compartment A (Lieberman-Aiden *et al.* 2009) under the facilitation of RYBP (RING1 and YY1-binding protein, enriched at Compartment A like CTCF, demonstrating high co-binding with CTCF on chromatin) to conduct phase separation beyond organizing the chromatin loops (Wei *et al.* 2022). The long-range chromatin interactions between Compartment A are likely to be organized by CTCF through non-canonical loop extrusion model (Wei *et al.* 2022).

**Figure 2:**
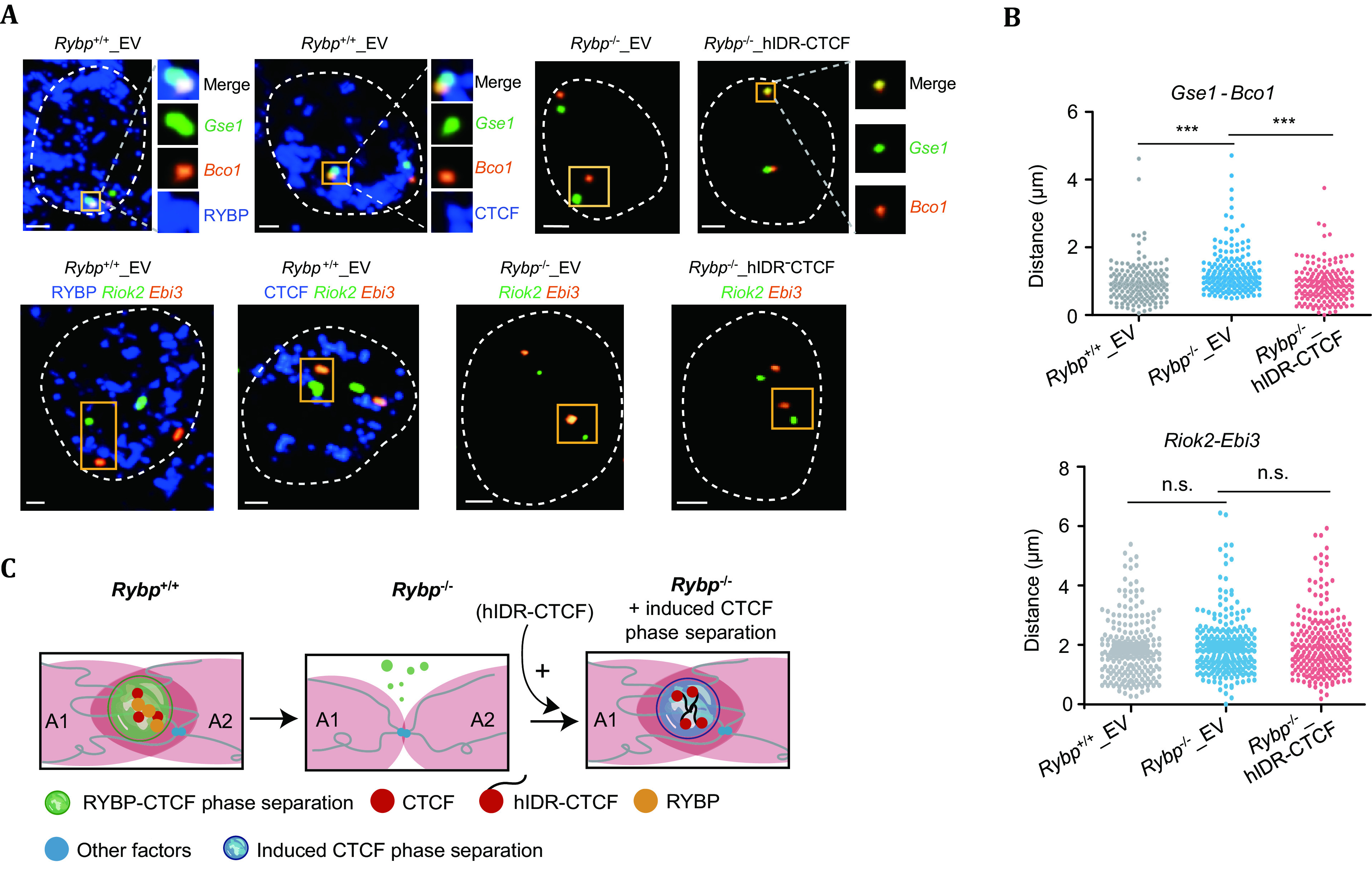
**A** *Gse1* and *Bco1*, *Uhrf1* and *Msh6* are paired genes in CTCF-enriched loci from different Compartment A. Through the employment of 3D-FISH, the change of distance of the paired gene can be imaged and examined. **B** Welch’s *t*-test on *Gse1* and *Bco1*, *Uhrf1* and *Msh6*. *Gse1* and *Bco1* obtained *p*-value of 5.12 × 10^-7^ and 5.782 × 10^-8^ (from left to right) suggesting distance increased upon the absence of *Rybp*. **C** A schematic demonstration of inter-compartment interactions under the presence and absence of *Rybp* along with a demonstration of induced CTCF phase separation in *Rybp* knocked out cell line (Distributed under the terms of the Creative Commons CC BY license, Wei *et al.* 2022)

FISH used in this work functioned as an imaging and validation method to demonstrate the change of distance between Compartment A amongst *Gse1* and* Bco1, Uhrf1* and *Msh6,* as shown in Fig. 2A. RYBP-Knockout (Rybp^-/-^) was produced to examine whether the RYBP would facilitate the aggregation of CTCF and to examine if depletion of RYBP would demonstrate any significant variation amongst the expression of CTCF. The results suggested the depletion of RYBP did disrupt the presentation of large CTCF puncta.

In Fig. 2, different cell lines (Fig. 2A) were used to demonstrate the distance between these paired genes, *Gse1* (Green) and *Bco1* (Yellow), increased upon the absence of RYBP (Rybp^-/-^), and decreased upon the presence of CTCF (Rybp^-/-^_hIDR-CTCF). The distance between two unpaired genes (*Riok2* and *Ebi3*) was not altered in RYBP-knockout cell lines which functioned as a negative control to cross examinate phase separation caused by CTCF induction. By offering sub-kilobase co-localization for the paired gene to reveal the change in distance, the hypothesis that induced CTCF or depleted RYBP would not affect inter-Compartment A interaction was validated.

#### lncRNA Platr22 regulation on nearby super-enhancers

Super-enhancers (SEs) are aggregate enhancers that harbor a high quantity of transcription factors along with mediator coactivators. Thus, SEs are vital in controlling the cell identity and disease state (Hnisz *et al.* 2013; Whyte *et al.* 2013). Longer than the typical enhancers (TEs), SEs demonstrated higher expressions of enhancer RNA, ten folds higher than TEs (Yan *et al.* 2020). LncRNAs demonstrated a tendency to preferentially locate next to Super Enhancers (SEs), suggesting that lncRNAs could be interacting with SEs. Knocking out SE-specific lncRNA transcripts leads to dysfunction of lncRNA activity (Yan *et al.* 2020). Pluripotency-associated transcript 22 (Platr22), a lncRNA gene, was demonstrated to regulate the expression of nearby SEs and other regulators such as ZFP281. Furthermore, Platr22 transcripts also interact with DDX5 (later recruits p300 for active transcription) and hnRNP-L, and Platr22 also coats chromatin SEs region. All these activities performed by Platr22 lead researchers to believe that Platr22 facilitates a transcription hub with these factors (Yan *et al.* 2020).

Thus, in the effort to reveal the relationship between nearby long noncoding sequences (lncRNA) and SEs, the comprehensive locus characterization was conducted in mouse embryonic stem cells (mESCs) to examine the SEs activities while SE-associated lncRNAs were knocked out.

**Figure 3:**
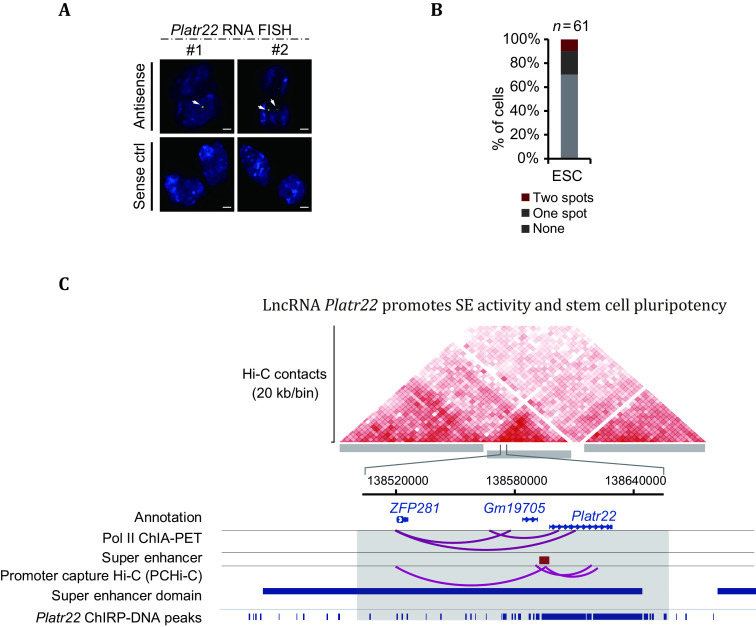
*Platr22* imaged using Tn5-based RNA-FISH on mESCs. **A** Upper panel: the Antisense. Bottom panel: sense control functioned as a control. In the antisense, one to two foci could be identified. The upper panel demonstrated how *Platr22* would interact solely within its own genomic region. **B** A statistical summary of embryonic stem cells contains either none, one, or two spots of *Platr22*. **C** A contact ability map based on Hi-C showing the interaction between genomic regions, and the results revealed that *Platr22*’s tendency to interact within its genomic loci (Distributed under the terms of the Creative Commons CC BY license, Yan *et al.* 2020)

RNA FISH was conducted on the *Platr22* transcript to demonstrate one or two foci in the nucleus (Fig. 3A), suggesting that the lncRNA transcripts are associated with nearby genomic locus predominantly (Yan *et al.* 2020). This experiment validated the researcher’s presumption on Platr22’s regulatory functionality of binding to their nearby SEs locus to facilitate an interaction transcription hub with ZFP281 and other factors (Fig. 3C).

### Tn5-based FISH in differentiated cells

#### Validating MyoD as a genome structure organizer in muscle cells

MyoD, a member of the myogenic regulatory factor family (Faerman *et al.* 1995; Megeney *et al*. 1996; Montarras *et al*. 2000; Ott *et al.* 1991; Sabourin *et al*. 1999; Sassoon *et al.* 1989; Tajbakhsh *et al.* 1996), functioned as a helix-loop-helix protein transcription factor. MyoD was found to function in critical process during early embryogenesis (Kassar-Duchossoy *et al*. 2004; Rudnicki *et al.* 1992, 1993). To further investigate the role of cell-lineage-specific master transcription factors and to disclose the functionality of MyoD in the genomic organization, researchers utilized wild-type and MyoD knocked-out (MKO) groups to demonstrate how MyoD functioned uniquely as a genomic organizer to mediate chromatin loops during muscle cell development (Wang *et al.* 2022).

**Figure 4:**
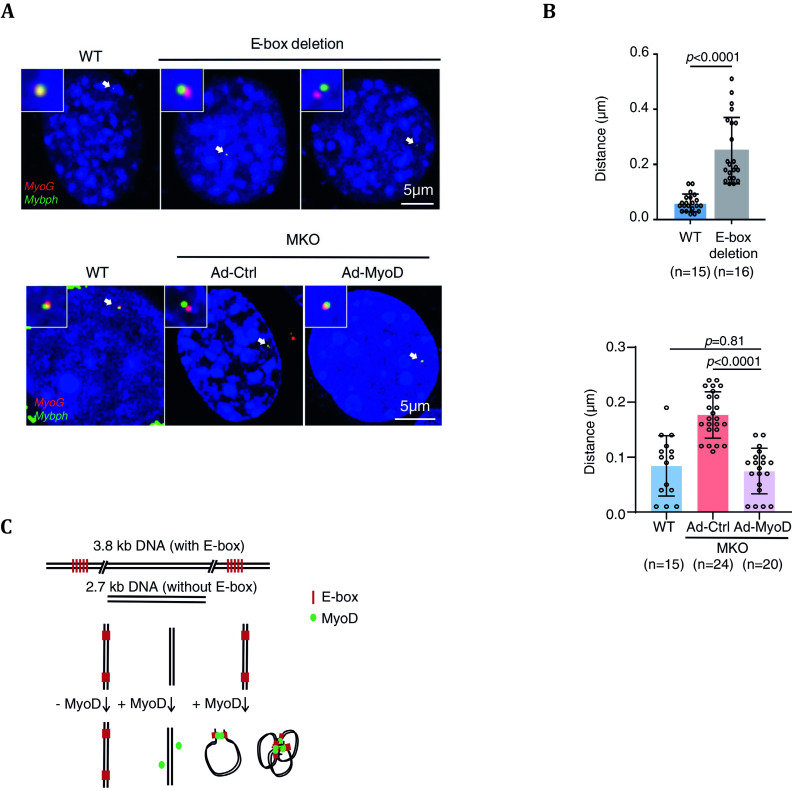
**A** Tn5-based DNA FISH utilized to examine the spatial distancing of the wild-type group compared with E-box deletion (upper panel) and MKO (lower panel). **B** Statistical analysis confirming the increase in spatial distancing between *MyoG* (red) and *Mybph* (green). **C** The schematic demonstration of how E-box and MyoD assist the folding of DNA (Distributed under the terms of the Creative Commons CC BY license, Wang *et al.* 2022)

In the wild-type group, the chromatin loop appeared to facilitate the interaction between two promoters since two Tn5-FISH dots (*MyoG* promoter (red) and *Mybph* promoter (green)) were tightly associated with each other. In E-box deleted and MKO groups, the two Tn5-FISH dots demonstrated an increase in spatial distance, suggesting MyoD was involved to mediate chromatin loop between two promoters (Fig. 4A). Furthermore, with statistical analysis, we demonstrated that the distance in wild-type group is lower than that in the MyoD knocked-out group (Fig. 4B). Through Tn5-FISH validation, researchers found that MyoD not only controls the A/B compartment organization in muscle cells, but also plays a vital role in myogenesis and formation of chromatin loops in muscle cells (Wang *et al.* 2022).

#### Validating spatial distance between different genomic loci in subcompartments

The integration of Hi-C and FISH data enables the localization and rationalization of the comprehensive spatial position of chromosomes. Intra-TAD reconstruction could be obtained from the information of the Hi-C contact map and previous biophysical knowledge of the polymer model. Coupling inter and intra-TAD reconstruction, the 3D comprehensive organization of chromosome conformation can be acquired, based on the computational integration of Hi-C and FISH data (Abbas *et al.* 2019). The method, GEM-FISH (Genomic orgranization reconstructor based on conformational Energy and Manifold learning-FISH), directly transfers nearby proximity of Hi-C data into spatial data (Abbas *et al.* 2019).

**Figure 5:**
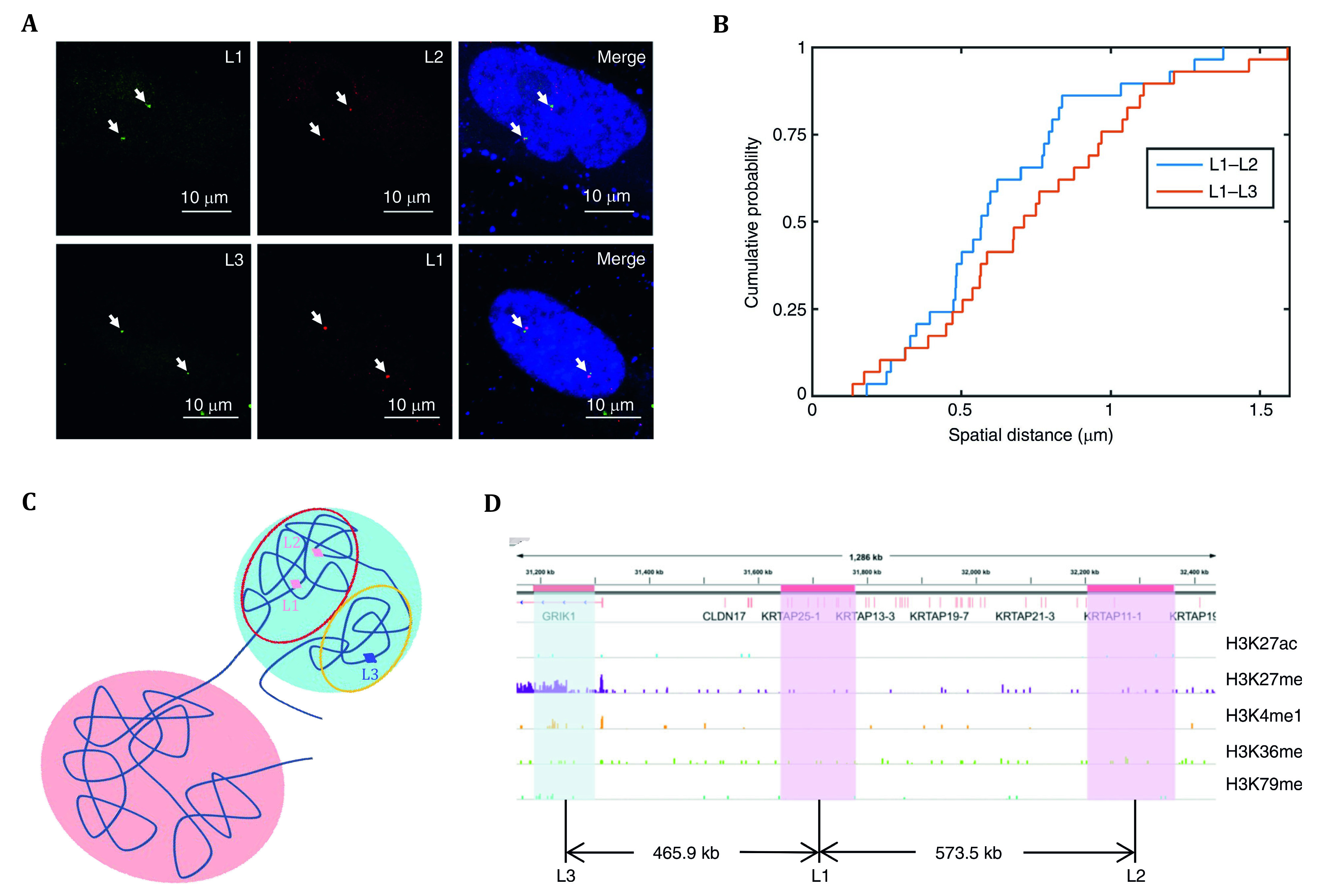
**A** The upper panel on the left demonstrated genomic loci L1 and L2 through DNA-FISH. L1 and L2 belong to Subcompartment B1. The bottom panel on the left demonstrated genomic L1 and L3 through DNA-FISH. L3 belongs to Subcompartment B2. **B** The cumulative distribution function (CDF) on the right shows the spatial distance of L1–L2 (blue line) is closer than L1–L3 (orange line) since L1 and L2 are in the same subcompartment (Abbas *et al.* 2019). **C** The blue region marks Subcompartment B, and the red region marks Subcompartment A. L1 and L2 located in Subcompartment B1 (the red hollow circle), and L3 located in the yellow hollow circle of Subcompartment B. L1 and L2 demonstrated to be spatially closer compared with L3. **D** Chi-seq data showing the distance of different histone marks of L1, L2, and L3 (Distributed under the terms of the Creative Commons CC BY license, Abbas *et al.* 2019)

To further validate the predictions made by GEM-FISH, three genomic loci probes were constructed to target sub-chromosomal level compartments (L1, L2 and L3). Genomic loci L1 and L2 belong to the same sub-chromosomal Compartment B1, while L3 belongs to Compartment B2 (Fig. 5A). FISH was used to examine the distance between L1–L2 and L1–L3, so that the result could cross-validate with the experimental evidence that loci in the same subcompartment are spatially closer than loci in different subcompartments. Through cumulative distribution function (CDF), FISH experiments show that the distance between L1–L2 (blue line) is indeed spatially closer than that between L1–L3 (orange line) (Fig. 5B), suggesting that image-based results are powerful to validate that L1 and L2 both belonged to Subcompartment B1, while L3 belonged to the B2 (Fig. 5C).

#### Tn5-FISH *application in leukemia cells*

5’-3’ loop and promoter-enhancer loop are the conventional type of chromatin loops that promote gene transcription (Cavalli and Misteli 2013). To examine the effect of gene-regulatory structure along with its relationship to gene expression in Leukemia, an ATRA (all-trans-retinoicacid)-induced HL-60 cell differentiation model was employed (Li *et al.* 2018).

**Figure 6:**
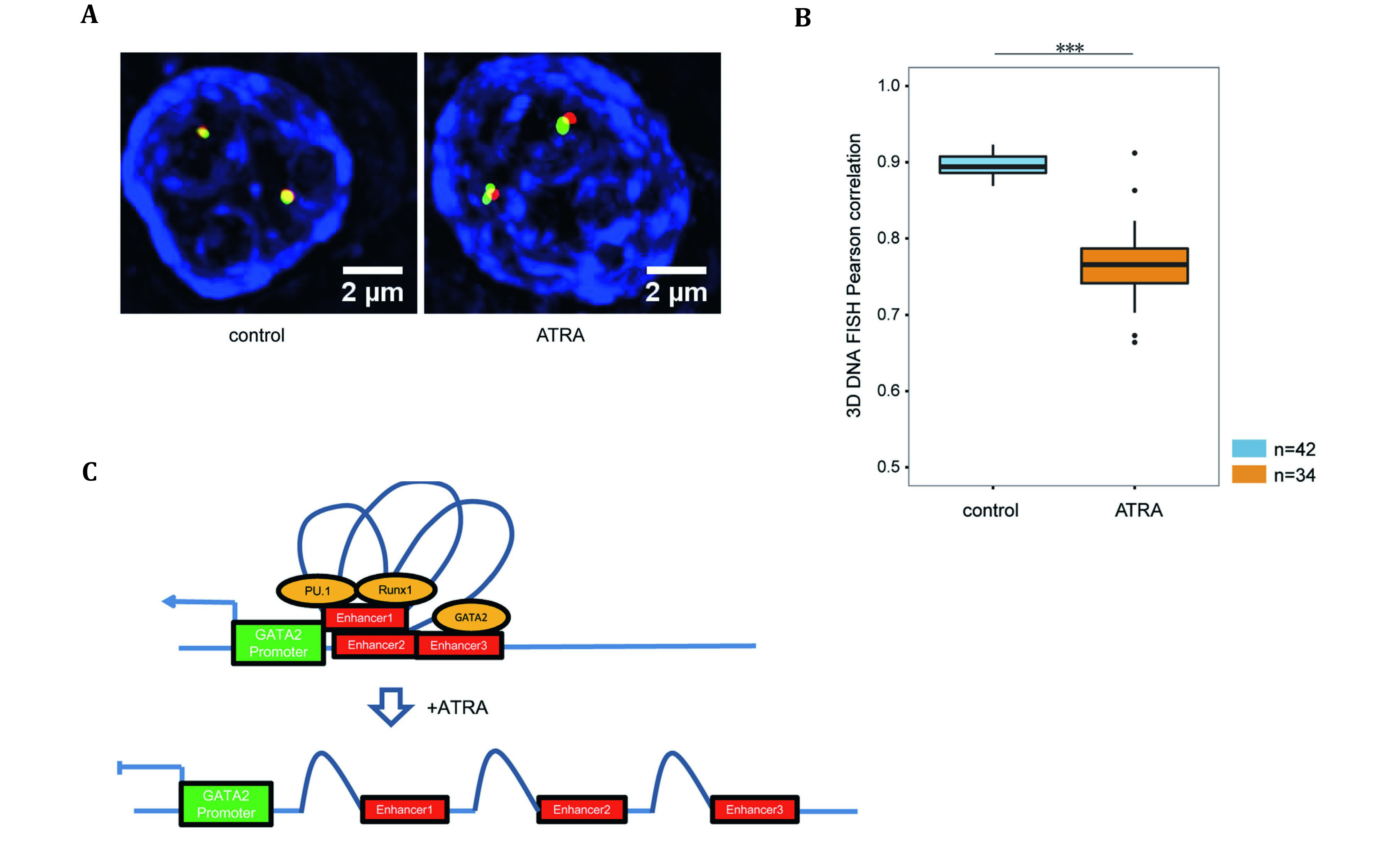
**A** The Tn5-based FISH showed that distances between GATA2 promoter (green) and enhancer (red) in the ATRA-treated group demonstrated to be longer than the control group. **B** Mann-Whitney *U*-test demonstrated the control group has more interactions between GATA2 promoters and enhancers than the ATRA-treated group. The visual illustration of GATA2 promoter and enhancers under ATRA-induction. **C** A demonstration of induced-ATRA would increase distances between GATA2 and other enhancers (Distributed under the terms of the Creative Commons CC BY license, Li *et al.* 2018)

Tn5-FISH provided visual confirmation that the distance between the GATA2 promoter and enhancer increased in the ATRA-treated group, compared with two congested fluorescence dots in the controlled group of intact chromatin loop (Fig. 6A), indicating that the distance between the GATA2 promoter and enhancer (Andersson *et al*. 2014) could be regulated by chromatin loops. A Pearson correlation between the controlled and ATRA-treated group provided statistical confirmation for the controlled group to be more interactive with promoter and enhancer (Fig. 6B). By this way, the relationship between chromatin conformation and transcription regulation was identified since the loss of chromatin loop structure around GATA2 would affect cell differentiation.

In another application, the FISH experiment showed that the fused gene t(9;11) (Mono-Mac6 cell line) and t(4;11) (MV4-11 and RS4;11 cell line) showed rearrangement in nuclear shell position, indicating that the difference in nuclear position and gene density after chromosomal translocations between normal and fused genes could possibly serve as a reason for the pathogenic role of leukemia cells (Murmann *et al.* 2005; Zhang *et al*. 2016). The spatial positioning of *MLL* (*m*ixed-*l*ineage-*l*eukemia or *m*yeloid-*l*ymophoid-*l*eukemia, now renamed as *KMT2A*) (Britten *et al*. 2019; Ziemin-van der Poel *et al*. 1991; Zotova *et al*. 2021) and *MLL*-related genes, including *AF4*, *AF6*, *AF9*, *ENL*, and *ELL* (the most common translocating partner of *MLL*), can be the possible contributing factors of human leukemias.

The findings could provide a new direction for pharmaceutical research on drug development.

## SUMMARY AND FUTURE PERSPECTIVE

From the perspective of genomics and transcriptomics, chromatin conformations and architectures are crucial to gene expression and regulation. In these applications discussed above, (Tn5-)FISH demonstrated its robust ability to couple experiments and functionality as a validation for sub-chromosomal level measurements in leukemia, mESCs, and differentiation cell lines.Tn5-FISH demonstrated its cogent usage in detecting genomic architectures and interactions, indicating that Tn5-FISH functioned as a powerful spatial imaging tool that could be used in scientific experiments and clinics by the researchers for validation on sub-chromosomal level targets.

From the perspective of the orchestra of multi-omics (including genomics, epigenomics, proteomics, lipidomics, glycomics and metabolomics), glycomics provides a new dimension of medical science–glycomedicine, under the hypothesis of “para-central dogma” (Wang 2022). Bioorthogonal chemical labelling enables to integrate Tn5-FISH and glycan imaging (Lu *et al*. 2023; Ma *et al*. 2023), thus holds tremendous potential to provide robust visualization evidence for glycan-protein interplay, glycan-RNA interactions and glycan-DNA interactions within cells and tissues.

On the other hand, Tn5- FISH has several limitations. It requires empirical design on the probe. Tn5-FISH performed less than expected when (1) probes against targeted sequence are less than one kilobase, and (2) probes are designed for low mappability regions. These existing limitations are expected to be resolved by elegant molecular approaches in the future.

In the era of spatial genomics, obtaining high-resolution pairwise distance information in a high-throughput manner remains challenging (Fudenberg and Imakaev 2017), even with the recent advancements in FISH methods (Beliveau *et al.* 2015; Boettiger *et al.* 2016; Fabre *et al.* 2015; Shachar *et al.* 2015). Despite all these, the multiplexed version of Tn5-FISH is currently under development. Moreover, by combining signal enhancement method (such as HRP, isothermal-amplification, ring-cycling amplification, *etc*.) and artificial intelligence, Tn5-FISH can achieve the kilobase-level global mapping of the chromatin structure in a high-throughput way.

## Conflict of interest

Liheng Yang, Yan Yan, JunLin Li, Cheng Zhou, Jinlan Jin, Tongmei Zhang, Haokaifeng Wu, Xingang Li, Wei Wang, Li Yuan, Xu Zhang and Juntao Gao declare that they have no conflict of interest.
